# Wnt Inhibition Sensitizes PD-L1 Blockade Therapy by Overcoming Bone Marrow-Derived Myofibroblasts-Mediated Immune Resistance in Tumors

**DOI:** 10.3389/fimmu.2021.619209

**Published:** 2021-03-15

**Authors:** Tinglei Huang, Fuli Li, Xiaojiao Cheng, Jianzheng Wang, Wenhui Zhang, Baiwen Zhang, Yao Tang, Qingli Li, Cong Zhou, Shuiping Tu

**Affiliations:** ^1^ State Key Laboratory of Oncogenes and Related Genes, Department of Oncology, Renji Hospital, School of Medicine, Shanghai Jiao Tong University, Shanghai, China; ^2^ Department of Oncology, the Affiliated Cancer Hospital of Zhengzhou University, Henan Cancer Hospital, Zhengzhou, China; ^3^ Shanghai Institute of Precision Medicine, Ninth People’s Hospital, Shanghai Jiao Tong University School of Medicine, Shanghai, China

**Keywords:** immunotherapy resistance, programmed cell death ligand 1, immune checkpoint blockers, cancer-associated fibroblasts, bone marrow-derived myofibroblasts, Wnt/β-catenin signaling pathway

## Abstract

Cancer-associated fibroblasts (CAFs) has been recognized as one cause of tumor resistance to immune checkpoint blockade therapy, but the underlying mechanisms still remain elusive. In the present study, a bone marrow-derived CAF (BMF) -rich tumor model is successfully established by subcutaneously mixed inoculation of BMFs and tumor cells into mice and the BMF-mixed tumor xenografts are demonstrated to be resistant to anti-PD-L1 antibody immunotherapy compared to the mere tumor xenografts. In vitro assays *via* the co-culture system of BMFs and tumor cells indicate that the co-cultured BMFs are induced to overexpress PD-L1, while there is no such a phenomenon in the co-cultured cancer cells. The further knock-out of PD-L1 in BMFs rescues the sensitivity of BMF-mixed tumor xenografts to PD-L1 blockade therapy. Mechanistically, *via* the microarray assay, we identify that the upregulation of PD-L1 in BMFs stimulated by cancer cells is medicated by the activation of the Wnt/β-catenin signaling pathway in BMFs. Moreover, the administration of Wnt/β-catenin signaling inhibitors, including XAV-939 and Wnt-C59, distinctly inhibits the upregulation of PD-L1 expression in the co-cultured BMFs. The further combination administration of XAV-939 significantly potentiates the therapeutic outcome of PD-L1 blockade therapy in BMF-mixed tumors. In summary, our study demonstrates that Wnt inhibition augments PD-L1 blockade efficacy by overcoming BMF-mediated immunotherapy resistance.

## Introduction

For the development of cancer therapy over the past decade, immunotherapy stands one of the most substantial breakthroughs and has revolutionized the cancer armamentarium (1). Cancer immunotherapy is a strategy to treat malignancies by leveraging the cytotoxic potential of the components of immune system, such as tumor-specific cytotoxic T cells. Among them, immune checkpoint blockade therapy, especially the inhibition of the axis of programmed cell death 1 (PD-1) and PD-1 ligand 1 (PD-L1), has achieved unprecedented success (2–4). The overexpressed PD-L1 on tumor cells serve to interact with the immune checkpoint molecule, PD-1, on activated T cells to cause dysfunction and exhaustion of cytotoxic T cells, thus escaping the immune surveillance to prevent the clearance of tumor cells. It has been demonstrated that the high expression of PD-L1 may account for the unfavorable clinical prognosis of the cancer patients (5, 6). Hence, immune checkpoint blockers (ICBs) designed to block the PD-1/PD-L1 interaction between tumors and activated T cells can inhibit tumor growth by enhancing antitumor immunity activity (7, 8). Nevertheless, emerging clinical evidences have revealed that response rate of patients to the PD-1/PD-L1 therapy is rather heterogeneous, with only less than 20% of the patients responding effectively to the immunotherapy of PD-1/PD-L1 (9–11).

In addition to the tumor cell intrinsic factors that enable immune-evasive capacity, tumor cell extrinsic factors including tumor-associated stroma and host immune system also have been corroborated to play a fundamental role in the ICBs therapy resistance (12). Cancer-associated fibroblasts (CAFs) are the major components of the tumor stroma and according to the tissue origin, CAFs can be categorized into bone marrow-derived myofibroblasts (BMFs), resident-tissue derived fibroblasts, vasculature system derived fibroblasts, and cancer cells derived fibroblasts (13). As indicated by our previous work, BMFs were functionally more capable of promoting tumor growth and invasion compared with normal or resident fibroblasts (14). It is demonstrated that BMFs can stimulate tumor malignant progression by contributing to the formation of mesenchymal stem cell niche or by inducing non-cancer stem cells (CSCs) to transform to CSC-like cells (14–16). Moreover, accumulating evidence has revealed that CAFs also dysregulate the immune microenvironment in different ways to cause tumor resistance to immunotherapy. For instance, CAFs secrete a large amount of TGF­β and support the formation of the desmoplastic stroma, which serves as a physical barrier to sequestrate immune cells (17). In addition, CAFs exhaust tumor infiltrating T cells by a contact dependent mechanism or exert immunosuppressive effects *via* releasing metalloproteinases to cleave NK cell–activating receptor ligands (1, 18, 19). However, whether the crosstalk between cancer cells and BMFs contribute to immunotherapy resistance and the possible mechanisms still entail further investigation.

Wnt/β-catenin signaling pathway has been identified to promote the initiation and expansion of many types of cancers, which could serve as one of the most-recognized molecular targets for cancer therapy (20, 21). However, a recent study has also shown that abnormal activation of Wnt/β-catenin pathway in tumor microenvironment is responsible for the failure of immunotherapy (22). In this study, we found that BMFs contributed to the anti-PD-L1 (aPD-L1) antibody immunotherapy resistance *via* the upregulated expression of PD-L1 in BMFs, which was mediated by the crosstalk between BMFs and cancer cells. Mechanistic study illustrated that tumor cells activated the Wnt/β-catenin signaling pathway to induce the PD-L1 overexpression in BMFs. Furthermore, the combination therapy with Wnt/β-catenin signaling inhibitor XAV-939 rescued the immunosuppressive status in the tumor microenvironment and enhanced the therapeutic efficacy of aPD-L1 therapy.

## Materials and Methods

### Cell Culture and Reagents

Mouse lung carcinoma cell line CMT167 and mouse colorectal cancer cell line MC38 were bought from ATCC. Bone marrow–derived myofibroblasts (BMFs, EGFP+) were isolated from gastric dysplastic tissues of EGFP+ bone marrow-transplanted mice in our laboratory. CMT167, MC38 and BMFs were cultured in RPMI-1640 media with 10% fetal bovine serum, 100 U/ml penicillin and 100 μg/ml streptomycin. BMFs within 10 passages were used for further assays. Mycoplasma negative was routinely confirmed in all cell lines. XAV-939 and Wnt-C59 were purchased from MCE (Shanghai, China). InVivo mAb anti-mouse PD-L1 (B7-H1) was purchased from Bio X Cell.

### Western Blotting

After the extraction of total protein using protein lysis buffer, the concentration of total protein was detected by the BCA method. The heat-denatured protein samples were then used for SDS-PAGE and electro-transferred to PVDF membrane, which was subsequently blocked by 5% skim milk for 1.5 h at room temperature (RT). Next, the membranes were incubated with specific primary antibodies at 4°C for 12 h, followed by three times of wash and incubation with corresponding secondary antibodies at RT for 1 h. The protein expression level was measured by ECL chemical luminescence. β-Actin was used as internal reference.

### Dual Luciferase Reporter Assay

The TOP-Flash or Fop-Flash reporter plasmids was transfected into BMFs cells, with Renilla luciferase reporter plasmid being co-transfected as standardized reference. 24 h post transfection, BMFs cells were seeded alone or co-cultured with tumor cells with or without the XAV-939 or Wnt-C59. 48 h later, the activity of the Firefly and Renilla luciferase in BMFs was determined using the Dual Luciferase Assay Kit (Promega). The Wnt-reporter activity was calculated as the ratio of Firefly luciferase activity to Renilla luciferase activity.

### The Harvest of PD-L1-Knockout BMFs Using the CRISPR/CAS9 System

CRISPR/CAS9 technique was used to knockout the endogenous PD-L1 gene in BMFs. Briefly, the guide sequence (5′-GTATGGCAGCAACGTCACGA-3′)targeting mouse PD-L1 genomic sequence were designed *via* CRISPR DESIGN (http://crispr.mit.edu/) and were cloned into the lenti-CRISPR vector plasmid. 48 h after transfection, cells were subcloned. 10 days later, the edition of the genomic sequence of PD-L1 were tested by DNA sequencing and the PD-L1 expression was detected by flow cytometry.

### Immunocytochemistry

BMFs were seeded on cover slips alone or co-cultured with CMT167 or MC38 cells for 48 h and then subjected to cellular immunofluorescence (IF) experiments. Cells were washed once by PBS, and then fixed using 4% paraformaldehyde for 15 min, followed by two times of PBS wash. Next, the samples were blocked with 5% BSA and 0.1% Triton X-100-containing PBS for 1 h at RT, and rinsed 3 times with PBS. Subsequently, cells were incubated with specific primary antibodies overnight at 4°C, and rinsed with PBS for 3 times. Then, cells were incubated with fluorescein-labeled secondary antibody at RT for 1 h in dark followed by PBS washes. The cells were mounted by Fluoroshield Mounting Medium with DAPI. The images were observed by a laser scanning confocal microscope (ZEISS LSM880).

### Tumor Xenograft Models and Anti-Tumor Studies

Eight-week-old female C57BL/6 mice were obtained from the animal center of Renji hospital and housed in the specific pathogen-free condition. All animal operations were carried out in compliance to the ethical guidelines licensed by Institution of Animal Care and Use Committee of Renji Hospital affiliated to Shanghai Jiaotong University, School of Medicine. To develop the Tumor xenograft models, 5*10^5^ CMT167 (or MC38) cells alone or mixed with 5*10^5^ BMFs or BMFs^PD-L1-KO^ were subcutaneously inoculated into the right flanks of mice, respectively. To assess the anti-tumor activity of aPD-L1 and XAV-939, tumor-bearing mice were randomly grouped (n=5) and dosed with isotype antibody (10 mg kg^-1^) as control, aPD-L1 (10 mg kg^-1^), and/or XAV-939 (5 mg kg^-1^) according to specific experimental requirements *via* intraperitoneal administration. Tumor volume and body weight of mice were measured every 2 days. Tumor volume was calculated according to the formula: V = L×W×W/2 (L, the long diameter; W, the shorter diameter) and the “tumor inhibitory rate” of the treatments was calculate as (V_Ctrl_ - V_Treated_)/V_Ctrl_. After the mice were sacrificed, tumor tissues were harvested to analyze the intratumoral infiltration of CD3^+^CD8^+^T cells and the fraction of CD8^+^IFN-γ^+^ cells *via* flow cytometry. In addition, tumor tissues were fixed, sectioned for immunofluorescence to detect the proportion of aSMA^+^ CAFs and CD8^+^ T cells. Peripheral blood serum samples were also collected to measure the serum levels of heart, hepatic, kidney function parameters. Major organs including lung, heart, liver and kidneys were also collected and sectioned for H&E staining to evaluate the biosafety of various treatments.

### Flow Cytometry

To determine the proportion of tumor infiltrated lymphocytes in tumors after various treatments, tumor tissues harvested from the sacrificed mice were spliced into small pieces and incubated in RPMI-1640 medium comprising 1 mg/ml collagenase IV and 0.2 mg/ml DNase I at 37°C for 45 min. The solution was then filtered through a 70 μm filter to acquire single cell suspension. After the viability staining and FcγR blocking, the cell suspension was stained with Fixable Viability Stain 510 (BD Biosciences), anti-CD45-PE-Cy7 (BD Biosciences), anti-CD3-BV650 (BD Biosciences), anti-CD8-FITC (BD Biosciences), and anti-IFN-γ-APC (BD Biosciences) according to manufacturer’s protocol. Flow cytometry was performed on BD LSR Fortessa (BD Biosciences) and analyzed using FlowJo (Tree Star) software.

To determine the expression level of different immunomodulatory ligands on BMFs and tumor cells, EGFP^+^BMFs and tumor cells were harvested and incubated with Fixable Viability Stain 780 (BD Biosciences), anti-PD-L1-APC (BD Biosciences), anti-CD70-PE(BD Biosciences), anti-CTLA-4-PE(BD Biosciences), anti-CD80-PE(BD Biosciences), anti-CD86-APC(BD Biosciences), anti-MHC-I-PE-Cy7 (eBioscience)and MHC-II-PE (BioLegend) antibodies according to manufacturer’s protocol. Flow cytometry was performed on CytoFLEX device (Beckman Coulter) and analyzed using FlowJo (Tree Star) software.

### Complementary Deoxyribonucleic Acid Microarray and Bioinformatics Analysis

Total RNA from BMFs cultured alone and BMFs co-cultured with tumor cells were extracted and subjected to Affymetrix microarray analysis as previously described (16). Genes with a fold change >1.5 and padj <0.05 were deemed as differentially-expressed gene and were conducted to KEGG pathway enrichment analysis in DAVID (https://david.ncifcrf.gov/).

### Statistical Analysis

All data were presented as mean ± standard deviation (SD). The statistical analysis was proceed using GraphPad software by two-tailed Student’s t-test for the comparisons between two groups and one-way analysis of variance (ANOVA) with Tukey’s *post hoc* analysis for multiple comparisons. Differences were considered statistically significant when P < 0.05 (*p < 0.05, **p < 0.01, ***p < 0.001, and ****p < 0.0001).

## Results

### BMFs Promote Tumor Resistance to aPD-L1 Immunotherapy by Suppressing Anti-Tumor Immune Response

**Figure 1:**
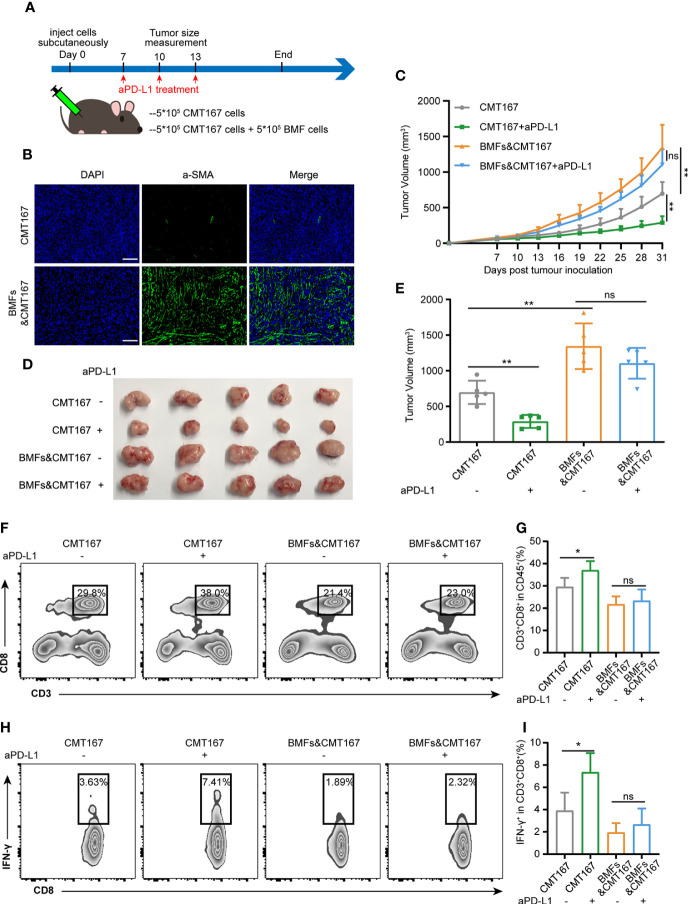
BMFs promote resistance to aPD-L1 immunotherapy by suppressing anti-tumor immune response. **(A)** Schematic diagram for the therapeutic regimen of anti-PD-L1 antibody in tumor-bearing mice injected subcutaneously with CMT167 cells (5*10^5^) alone or mixed with BMFs (5*10^5^). **(B)** Representative immunofluorescence staining of aSMA (green) in tumor samples implanted with CMT167 cells alone or CMT167 cells mixed with BMFs. DAPI was stained to visualize cell nuclei. Scale bar = 100 μm. **(C–E)** Average tumor growth curves **(C)**, the images of tumor tissue dissected from animals **(D)** as well as individual tumor volume **(E)** at the end of the study. **(F–I)** Representative flow cytometry plots **(F, H)** and quantitative analysis **(G, I)** of tumor infiltration CD3^+^CD8^+^ and CD8^+^ IFN-γ^+^ subsets. The data were plotted as means ± SDs (n = 5 per group) and analyzed using two-tailed Student’s t-test for the comparisons between two groups and one-way analysis of variance (ANOVA) with Tukey’s *post hoc* analysis for multiple comparisons. Differences were considered statistically significant when P < 0.05 (*p < 0.05, **p < 0.01, ns stands for not significant).

To investigate the effect of BMFs exerted on the PD-L1 blockade therapy, BMF-rich mouse tumor model was developed by subcutaneously inoculation of the mixture of BMFs and murine lung carcinoma cell CMT167 to the flanks of mice, followed by various treatments to the mice with isotype control antibody or monoclonal aPD-L1, respectively, as illustrated in **Figure 1A**. Immunofluorescence (IF) staining images in **Figure 1B** showed that the tissue sections from mice inoculated with cell mixtures of CMT167 cells and BMFs showed significantly higher quantity of aSMA^+^ fibroblasts compared with the mice inoculated with CMT167 cells alone. Moreover, the BMF-mixed tumor group exhibited significantly larger tumor size than the mere CMT167 tumor cell group (**Figures 1C–E**). Meanwhile, aPD-L1 treatment robustly alleviated the growth of tumor inoculated with CMT167 cells alone, whereas there was no significant tumor shrinkage in aPD-L1 treated BMF-rich tumors (**Figures 1C–E**). Similar results were obtained for the aPD-L1 treatment in subcutaneous MC38 colorectal cancer mouse model co-inoculated with BMFs or not (**Supplementary Figure 1**).

To evaluate the anti-tumor immune responses underlying the resistance of aPD-L1 therapy in BMF-rich tumors, the proportion of tumor infiltrated lymphocytes (TILs) in various tumors were analyzed by flow cytometry. aPD-L1 pronouncedly elevated the fraction of CD8^+^ T cells to 37% and that of CD8^+^IFN-γ^+^ T cells to 7.4% relative to the untreated group in the tumors injected with CMT167 cells alone (**Figures 1F–I**). However, BMF-mixed tumors with aPD-L1 immunotherapy exhibited low fractions of the infiltrating CD8^+^ T cells and CD8^+^IFN-γ^+^ T cells, which were comparable to that of the corresponding untreated group (**Figures 1F–I**). The above results indicated that BMFs significantly promoted the resistance to the PD-L1 blockade immunotherapy at least partly by suppressing the anti-tumor immune responses in the BMF-rich tumor mass.

### BMFs Enhance Tumor Progression by Attenuating Anti-Tumor Immune Responses in a PD-L1 Dependent Manner

**Figure 2:**
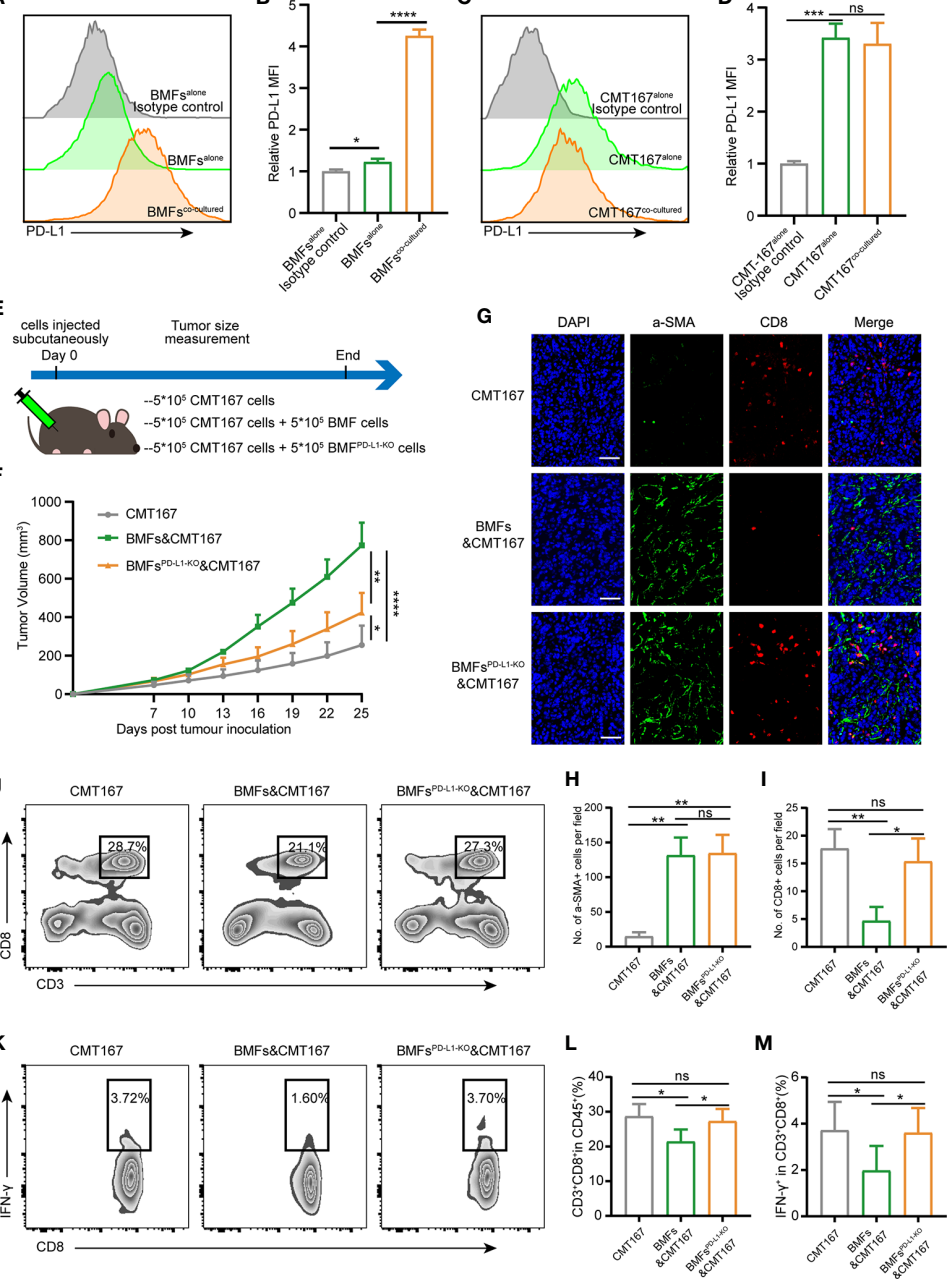
BMFs enhance tumor progress by attenuating anti-tumor immune responses in a PD-L1 dependent manner. **(A, B)** Representative histograms **(A)** and quantitative analysis **(B)** displaying the expression level of PD-L1 in BMFs cultured alone or co-cultured with CMT167. The data were plotted as means ± SDs (n = 3 per group). MFI, mean fluorescence intensity. **(C, D)** Representative histograms **(C)** and quantitative analysis **(D)** displaying the expression level of PD-L1 in CMT167 cultured alone or co-cultured with BMFs. The data were plotted as means ± SDs (n = 3 per group). MFI, mean fluorescence intensity. **(E)** Schematic diagram for the tumor subcutaneous xenograft model of CMT167 cells (5*10^5^) injected alone or mixed with BMFs (5*10^5^) or BMFs^PD-L1-KO^ (5*10^5^). **(F)** Average tumor growth curves from animals of the study. The data were plotted as means ± SDs (n = 5 per group). **(G–I)** Representative immunofluorescence staining image **(G)** and quantitative analysis **(H, I)** of aSMA^+^ (green) and CD8^+^ (red) cells in tumor samples injected with CMT167 cells alone or mixed with BMFs or BMFs^PD-L1-KO^. DAPI was stained to visualize cell nuclei. Scale bar = 50μm. **(J–M)** Representative flow cytometry plots **(J, K)** and quantitative analysis **(L, M)** of tumor infiltrating CD3^+^CD8^+^ and CD8^+^IFN-γ^+^ T cells. The data were plotted as means ± SDs (n = 5 per group). The data were analyzed using two-tailed Student’s t-test for the comparisons between two groups and one-way analysis of variance (ANOVA) with Tukey’s *post hoc* analysis for multiple comparisons. Differences were considered statistically significant when P < 0.05 (*p < 0.05, **p < 0.01, ***p < 0.001, and ****p < 0.0001. ns stands for not significant).

To clarify the mechanism by which BMFs contributed to the compromise of anti-tumor immunity conferred by the aPD-L1 therapy, the expression of a series of immunomodulatory ligands (including CD70, CTLA-4, CD80, CD86, MHC-I, MHC-II, and PD-L1) on the surfaces of BMFs and tumor cells was analyzed by flow cytometry. Among all the detected immunomodulatory ligands (**Supplementary Figure 2**), the expression level of PD-L1 on BMFs was relatively low when cultured alone, however, it was significantly upregulated in BMFs after their co-culture with various tumor cells (**Figures 2A, B**, **Supplementary Figure 3**). The expression of PD-L1 on the surface of tumor cells remained unchanged after the co-culture (**Figures 2C, D** **)**.

To figure out the role of upregulated PD-L1 in BMFs induced by tumor cells, PD-L1 of BMFs was knocked-out by CRISPR-CAS9 (**Supplementary Figure 4**). Then CMT167 cells alone and the mixture of CMT167 cells with BMFs or BMFs^PD-L1-KO^ were subcutaneously inoculated to the flanks of C56BL/6 mice, respectively, as illustrated in **Figure 2E**. Although both BMFs and BMFs^PD-L1-KO^ significantly promote the growth of tumors, the loss of PD-L1 expression in BMFs still distinctly diminished the protumorigenic capacity relative to the unmodified BMFs (**Figure 2F**, **Supplementary Figure 5**). IF staining results displayed that the depletion of PD-L1 in BMFs had no apparent effect on the proportion of aSMA^+^ BMFs in tumor tissues (**Figures 2G, H** **)**. Next, the flow cytometric analysis and IF staining results of TILs consistently demonstrated that the inhibitory effects of BMFs on the infiltration of CD8^+^ T cells and CD8^+^IFN-γ^+^ T cells in BMF-rich tumors were both notably rescued by the knock-out of PD-L1 expression in BMFs (**Figures 2G, I–M**).

### Knockout of PD-L1 in BMFs Potentiates the PD-L1 Blockade Immunotherapy in BMF-Mixed Tumors

**Figure 3:**
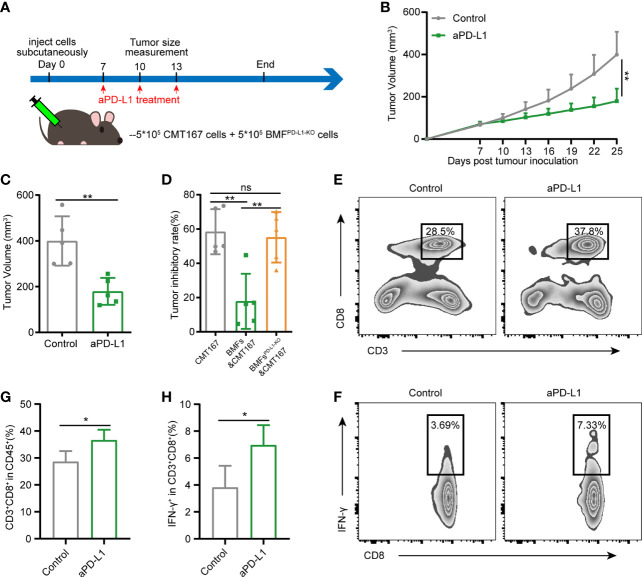
Knockout of PD-L1 in BMFs potentiates the PD-L1 blockade immunotherapy in BMF-rich tumors. **(A)** Schematic diagram for the therapeutic regimen of anti-PD-L1 antibody in tumor-bearing mice subcutaneously injected with CMT167 cells (5*10^5^) mixed with BMFs^PD-L1-KO^ (5*10^5^), followed by treatments with isotype control antibody or aPD-L1 antibody. **(B, C)** Average tumor growth curves**(B)** and individual tumor volume **(C)** of tumor tissue dissected from animals at the end of the study. **(D)** The tumor inhibitory rate of aPD-L1 immunotherapy in tumor xenografts implanted with CMT167 alone, CMT167 mixed with BMFs and CMT167 mixed with BMFs^PD-L1-KO^. **(E–H)** Representative flow cytometry plots **(E, F)** and quantitative analysis **(G, H)** of tumor infiltration CD3^+^CD8^+^ and CD8^+^ IFN-γ^+^ T cell. The data were plotted as means ± SDs (n = 5 per group) and analyzed using two-tailed Student’s t-test for the comparisons between two groups and one-way analysis of variance (ANOVA) with Tukey’s *post hoc* analysis for multiple comparisons. Differences were considered statistically significant when P < 0.05 (*p < 0.05, **p < 0.01, ns stands for not significant).

To assess the role of PD-L1 on BMFs in promoting the resistance to aPD-L1 immunotherapy, we subcutaneously injected cell mixtures of CMT167 cells and BMFs^PD-L1-KO^ into the flank regions of each mouse and treated them with aPD-L1 or control IgG as illustrated in **Figure 3A**. With the depletion of PD-L1 in BMFs, the immunotherapy of aPD-L1 again exhibited overt therapeutic outcome by significantly suppressing the growth of BMFs^PD-L1-KO^-rich tumors compared with those received control IgG (**Figures 3B, C**, **Supplementary Figure 6**). From the analysis of the tumor inhibitory rate of aPD-L1 immunotherapy in various tumors, which were shown in **Figures 1C–E** and **Figures 3B, C**, **Supplementary Figure 6** (CMT167 alone, CMT167 mixed with BMFs and CMT167 mixed with BMFs^PD-L1-KO^), the immunotherapy in BMFs^PD-L1-KO^-mixed tumor xenografts had an inhibitory rate of 55.1%, which was similar to that in pure CMT167 tumors (58.4%) and remarkably higher than that in BMFs- mixed tumor xenografts (17.8%), indicating that the knockout of PD-L1 from BMFs almost completely reversed the aPD-L1 immunotherapy-resistant effects of BMFs in BMFs-rich tumors (**Figure 3D**). Flow cytometric results of the TILs showed that aPD-L1 immunotherapy distinctly elevated the ratios of CD8^+^ T cells and CD8^+^IFN-γ^+^ T cells in the BMFs^PD-L1-KO^-mixed tumor xenografts as compared with the control group (**Figures 3E–H**).

### Tumor Cells Spur the PD-L1 Expression in BMFs by Stimulating Wnt/β-Catenin Signaling Pathway

**Figure 4:**
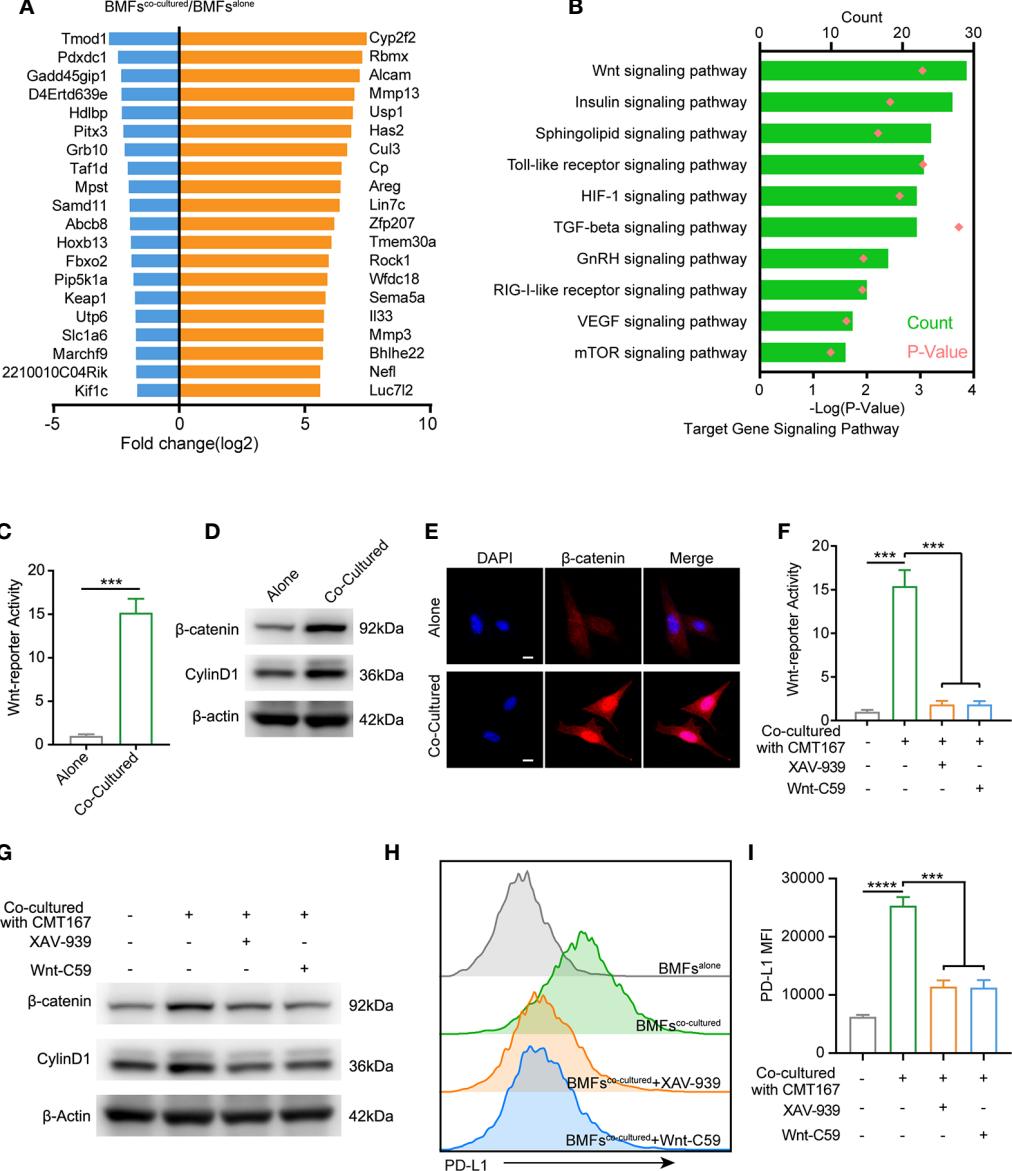
Tumor cell spur the PD-L1 express of BMFs by stimulating wnt/β-catenin signaling pathway. **(A)** The 20 most up-regulated and down-regulated genes from the microarray results of BMFs co-cultured with tumor cells vs. BMFs cultured alone. **(B)** Gene ontology analysis indicates significantly changed canonical pathways in BMFs after the co-culturing with tumor cells, (DAVID Bioinformatics Resources 6.8). **(C)** Relative Wnt reporter activity of BMFs cells cultured alone or co-cultured with CMT167. **(D)** Representative immunoblotting result of β-catenin and CylinD1 in BMFs cultured alone or co-cultured with CMT167. β-actin was used as the loading control. **(E)** Representative image of β-catenin (red) immunofluorescence staining in BMFs cultured alone or co-cultured with CMT167. DAPI was stained to visualize cell nuclei. Scale bar = 10 μm. **(F–I)** Relative Wnt reporter activity **(F)**, representative immunoblotting result of b-catenin, CylinD1 and b-actin **(G)** and PDL1 express level measured by flow cytometry **(H, I)** in BMFs cells cultured alone or co-cultured with CMT167 while the co-cultured group treated with 939(1 mM)/Wnt-C59(5 mM) or not. MFI, mean fluorescence intensity. Representative results were from one of at least three independent experiments. The data were plotted as means ± SDs (n = 3 per group) and analyzed using two-tailed Student’s t-test for the comparisons between two groups and one-way analysis of variance (ANOVA) with Tukey’s *post hoc* analysis for multiple comparisons. Differences were considered statistically significant when P < 0.05 (***p < 0.001, and ****p < 0.0001).

To further identify the underlying mechanism by which tumor cells boosted PD-L1 expression in BMFs, we performed microarray analysis to compare the transcriptomes in BMFs cultured alone or co-cultured with tumor cells. The top 20 differentially-expressed genes in BMFs after co-culture with tumor cells are listed in **Figure 4A**. KEGG analysis of transcriptomes revealed that differentially-expressed genes between BMFs cultured alone and BMFs co-cultured with tumor cells could be categorized into 10 signaling transduction pathways, among which the ‘‘Wnt signaling pathway’’ was the mostly-affected canonical pathway (**Figure 4B**). We further confirmed this result by Wnt-reporter assays, displaying that the tumor cells, indeed, activated the Wnt/β-catenin signaling pathway in the co-cultured BMFs (**Figure 4C**, **Supplementary Figure 7A**). Western blotting results demonstrated that the protein expression levels of β-catenin and Cyclin D1 (Wnt/β-catenin target gene) in the cocultured BMFs were both obviously upregulated compared to their counterparts in BMFs cultured alone (**Figure 4D**, **Supplementary Figure 7B**). Moreover, IF staining images of β-catenin in BMFs demonstrated that there was much more β-catenin localized in the nucleus of BMFs co-cultured with tumor cells, whilst there was only weak cytoplasmic β-catenin staining signal observed in BMFs cultured alone (**Figure 4E**, **Supplementary Figure 7C**).

We next assessed the effects of the selective inhibitors of Wnt/β-catenin signaling, including XAV-939 (a potent tankyrase inhibitor that targets Wnt/β-catenin signaling) and Wnt-C59 (a potent porcupine inhibitor), on the upregulation of PD-L1 in BMFs induced by CMT167 or MC38 cancer cells. Wnt-reporter assays and western blotting demonstrated that XAV-939 and Wnt-C59 effectively abrogated the activation of Wnt/β-catenin signaling in the co-cultured BMFs (**Figures 4F, G**, **Supplementary Figures 8A, B**). Meanwhile, flow cytometric analysis revealed that the administration of Wnt/β-catenin signaling inhibitors significantly reduced the PD-L1 expression on BMFs induced by tumor cell co-culture conditions (**Figures 4H, I**, **Supplementary Figures 8C, D**).

### Inhibition of Wnt/β-Catenin Signaling Boost the Efficacy of aPD-L1 Immunotherapy in BMF-Mixed Tumors

**Figure 5:**
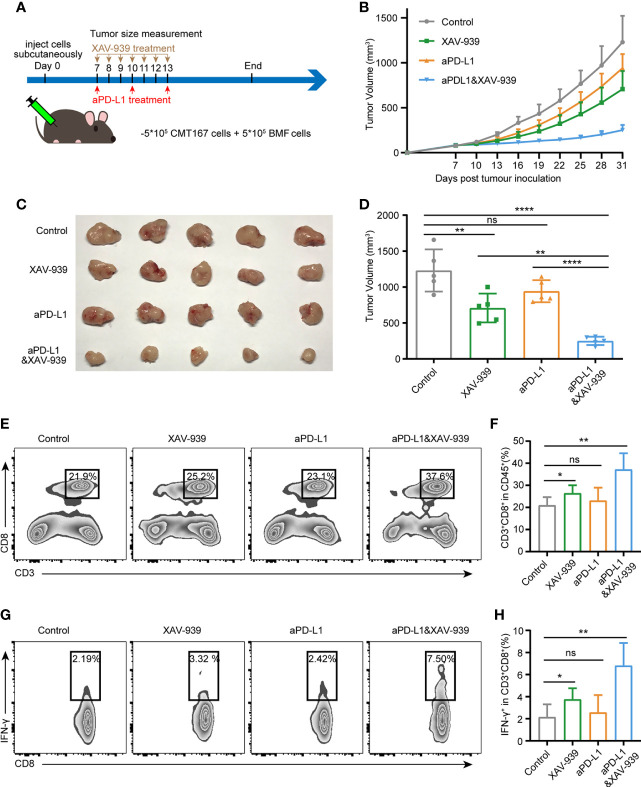
Wnt/β-catenin inhibitor XAV-939 boost the efficacy of aPD-L1 immunotherapy in BMF-rich tumors. **(A)** Schematic diagram for the therapeutic regimen of aPD-L1 antibody combined with XAV-939 in tumor-bearing mice injected subcutaneously with CMT167 cells (5*10^5^) mixed with BMFs (5*10^5^). **(B–D)** Average tumor growth curves **(B)**, the images of tumor tissue dissected from animals **(C)** as well as individual tumor volume **(D)** at the end of the study. **(E–H)** Representative flow cytometry plots **(E, G)** and quantitative analysis **(F, H)** of tumor infiltration CD3^+^CD8^+^ and CD8^+^ IFN-γ^+^ T cell. The data were plotted as means ± SDs (n = 5 per group). The data were analyzed using two-tailed Student’s t-test for the comparisons between two groups and one-way analysis of variance (ANOVA) with Tukey’s *post hoc* analysis for multiple comparisons. Differences were considered statistically significant when P < 0.05 (*p < 0.05, **p < 0.01, ***p < 0.001, and ****p < 0.0001. ns stands for not significant).

To investigate whether the combination use of the Wnt signaling inhibitor XAV-939 with aPD-L1 can improve the efficacy of aPD-L1 in BMFs-rich tumors, aPD-L1 and/or XAV-939 were treated to mice subcutaneously bearing BMF-mixed tumors as illustrated in **Figure 5A**. From **Figures 5B–D**, XAV-939 monotherapy significantly suppressed the tumor growth while the aPD-L1 monotherapy only displayed slight inhibitory effects on the tumor progression. Notably, the combined administration of XAV-939 with aPD-L1 treatment remarkably boosted the therapeutic effect of aPD-L1. Moreover, the flow cytometric results demonstrated that the combination use of XAV-939 and aPD-L1 significantly elevated the proportion of CD8^+^ T cells to 37% and CD8^+^IFN-γ^+^ T cells to 6.8% in the BMF-rich tumor microenvironment (**Figures 5E–H**), with only 23% CD8^+^ T cells and 2.6% CD8^+^IFN-γ^+^ T cells infiltrated into BMF-rich tumors after aPD-L1 monotherapy.

**Figure 6:**
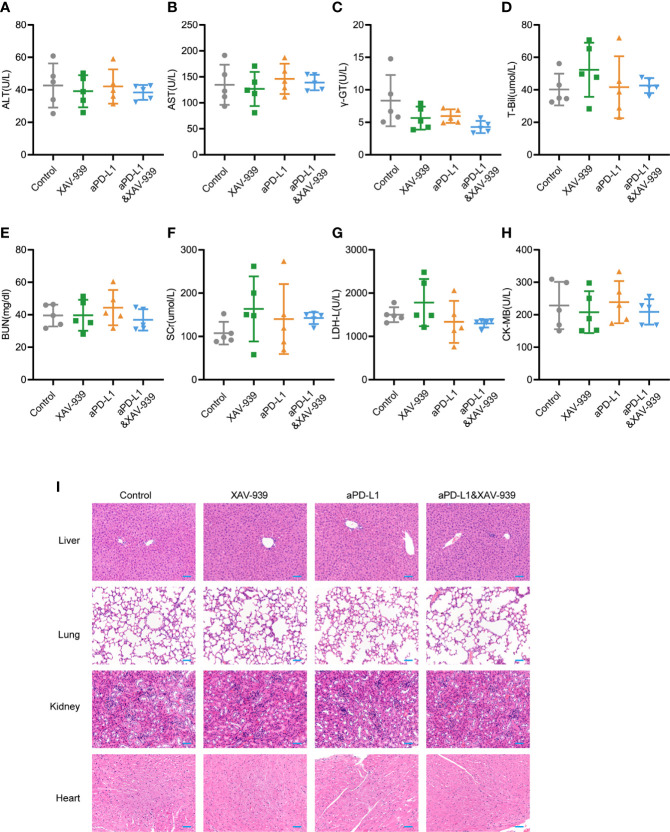
Safety evaluation of therapeutic regimen of aPD-L1 antibody combined with XAV-939. **(A–H)** The level of ALT **(A)**, AST **(B)**, r-GT **(C)**, T-Bil **(D)**, BUN **(E)**, SCr **(F)**, LDH-L **(G),** and CK-MB **(H)** in the serum of tumor-bearing mice at the endpoint of the therapeutic study illustrated in **Figure 5A** (n = 5, data presented as means ± SDs). **(I)** Representative H&E-stained images of heart, kidney, liver and lung from mice of each group at the endpoint of the therapeutic study illustrated in **Figure 5A**. Scale bar = 50 µm.

Since the biosafety issue is of great importance to the clinical translation of pharmaceuticals, the *in vivo* biosafety of current combination treatment strategy was also investigated by monitoring various physiological indicators of mice during the study. From **Supplementary Figure 9**, the body weight of mice receiving aPD-L1 and/or XAV-939 treatment remained largely stable throughout the treatment period. At the end of the study, the serum indexes of heart, liver and kidney function were all within the reference values (**Figures 6A–H**). Histological analysis of major organs *via* hematoxylin and eosin (H&E)-staining exhibited that there was no obvious damage among all treatment groups (**Figure 6I**).

## Discussion

Despite the immunotherapy regime, particularly the use of ICBs has revolutionized the anti-tumor treatment strategies, most of the patients (~80%) have different degree of resistance to the ICBs immunotherapy and the underlying mechanism of immunotherapy resistance still entails deeper investigation (9–11). Here, BMFs were demonstrated to suppress anti-tumor immune response to induce tumor resistance to aPD-L1 immunotherapy at least partly by the overexpressed PD-L1 expression on the surface of BMFs. Further study revealed that the crosstalk of BMFs with cancer cells induce the upregulation of PD-L1 expression in BMFs by activating the Wnt/β-catenin signaling pathway, which may be a potential therapy target to resensitizing BMF-rich tumors to aPD-L1 immunotherapy. Notably, we demonstrated that the inhibition of Wnt/β-catenin signaling pathway significantly abrogated the upregulation of PD-L1 in BMFs stimulated by cancer cells and remarkably boosted the therapeutic effects of aPD-L1 treatment by overcoming the immunosuppressive status in BMF-rich tumors.

By subcutaneous inoculation of mixtures of BMFs and tumor cells into mice, we successfully introduced BMFs into tumor microenvironment and established a BMF-rich tumor model. As illustrated in **Figure 1** and **Supplementary Figure 1**, BMFs significantly promoted tumor resistance to the aPD-L1 immunotherapy at least partially by restraining the anti-tumor immune response in the BMF-rich tumors. Recently, the role of CAFs in promoting immunotherapy resistance was demonstrated by Fatima Mechta-Grigoriou et al, indicating that myofibroblasts clusters characterized by extracellular matrix proteins and TGF-β signaling were correlated with primary resistance to immunotherapies *via* the positive feedback loop between the specific CAF clusters and regulatory T cells (23). It has also been reported that CAFs can indirectly suppress anti-tumor immunity by increasing PD-L1 expression in melanoma, colorectal cancer as well as lung adenocarcinoma cells (24, 25). To further investigate the mechanism underlying the resistance to aPD-L1immunotherapy in tumors with enriched amount of BMFs, *in vitro* co-culture system of various cancer cells and BMFs was adopted to detect the effects of cellular crosstalk on the expression of various immunomodulatory ligands on the surface of BMFs and tumor cells, respectively. Among all the detected ligands on BMFs and tumor cells after co-culture conditions, only the BMFs after the co-culture with tumor cells exhibited significantly upregulated expression level of PD-L1 (**Figures 2A, B**, **Supplementary Figure 3**), implying that the induction of PD-L1 on BMFs by cancer cells could account for the aPD-L1 therapy resistance in BMF-mixed xenografts. It is widely-accepted that normal fibroblast population is one of the most common cell types in normal tissues, responsible for the proper cell function and tissue homeostasis. Compared with normal fibroblasts, CAFs tend to accumulate in tumors tissues and can be activated by the interaction between fibroblasts and tumor cells or immune cells in the tumor microenvironment. Once activated, fibroblasts alter its biology and developed into CAFs, playing important roles in the development of tumor microenvironment (26). Hence, there could also exist a crosstalk between tumor cells and BMFs in this study, as the upregulated PD-L1 in BMFs could be triggered by tumor cells and BMFs seem to reciprocally decrease the immune infiltration into tumors to cause immunotherapy resistance.

In order to ascertain the role of the upregulated PD-L1 on BMFs in mediating the aPD-L1 immunotherapy resistance in BMF-rich tumors, PD-L1-knocked-out-BMFs were used to develop BMF^PD-L1-KO^-rich tumors in comparison with BMF-rich tumors. In vivo therapeutic results the knockout of PD-L1 in BMFs distinctly reduced the tumor-promoting capacity compared to the unmodified BMFs as well as rescued the inhibitory effects of BMFs on the anti-tumor immune responses, indicating that the BMFs induced facilitated-tumor growth and repressed-anti-tumor immune responses was, to some extent at least, caused by the upregulation of PD-L1 in BMFs. Meanwhile, the tumor-inhibitory effects and cytotoxic immune infiltration after aPD-L1 immunotherapy in BMFs^PD-L1-KO^-rich tumors consistently recovered to desirable levels, further validating the potential of targeting at the overexpressed PD-L1 in BMFs to overcome aPD-L1 therapy resistance.

Although increasing evidence have shown that patients with PD-L1-positive tumors have a higher response rate than PD-L1 negative patients in different cancer types, there are also reports claiming that many patients with PD-L1 positive tumors showing resistance to anti-PD-1/PD-L1 treatments (17, 27–29). Recently, Ryohei Katayama et al. have identified two secreted PD-L1 splicing variants in patients’ tumors relapsed from PD-L1 blockade therapy. These stably secreted PD-L1 variants can functioned as “decoys” to PD-L1 blockade antibodies and caused resistance to aPD-L1 blockade therapy (30). Moreover, the constitutive expression of the PD-L1 in tumor cells always accompanied with low level of TIL in tumor microenvironment, which formed the so-called immune-excluded or immune-desert phenotypes and was connected with restrained sensitivity to immunotherapy (1, 31). These evidences indicated that the persistent, uncleanable expression of PD-L1 from various sources in the tumor microenvironment could be a mechanism for the low responsiveness of aPD-L1 immunotherapy. The present study demonstrated that the overexpressed PD-L1 in BMFs played a vital role in the resistance to aPD-L1 monotherapy in BMFs-rich tumors, which indicated that the PD-L1 in BMFs may be a potential combination therapy target to improve the curative effect of aPD-L1 in BMFs-rich tumors.

According to recent progress of cancer immunotherapy, in addition to the blockade of PD-L1/PD-1 interaction, the inhibition of PD-L1 expression in cancer cells constitutes an alternative to augment the antitumor immunity activity and further inhibited tumor growth (7, 8, 32). It has been reported that PD-L1 expression can be modulated by several signaling pathways correlated to the genome, transcriptional or post-transcriptional activities as well as translational or post-translational events. Our previous studies have shown that the crosstalk between BMFs and tumor cells can regulate several critical oncogenic signaling pathways (14–16). In this study, *via* microarray assay, we found that tumor cells induced the upregulation of the PD-L1 expression in BMFs by activating the Wnt/β-catenin signaling pathway, providing a potential therapeutic target to overcome the resistance to aPD-L1 immunotherapy in BMFs-rich tumors. Accumulating clues indicated that deregulated Wnt signaling executive protumoral functions not only endowed tumor cells with malignant features (33–35), but also contributed to the invasion of anticancer immunosurveillance to induce resistance to multiple immunotherapeutic (36). Therefore, intervention of the Wnt/β-catenin signaling pathway could also be a promising perspective to improve the efficacy of immunotherapy. For instance, inhibiting Wnt/β-catenin signaling pathway is expected to enhance the efficacy of immunotherapy by upregulating tumor antigens release (37), enhancing DC antigen presentation and T cell priming (38–40), facilitating CTLs/Th cells infiltration in tumor microenvironment (41, 42). Meanwhile, it has been reported that the blockade of Wnt/β-catenin signaling has the potential to improve antitumor T cell responses *via* depleting immune checkpoint PD-1/PD-L1 in several kinds of tumor cells (43–46). In this work, we found that the crosstalk between cancer cells and BMFs remarkably upregulated the PD-L1 expression in BMFs by activation the Wnt/β-catenin signaling, meanwhile the inhibition of Wnt/β-catenin signaling pathway can significantly suppress the PD-L1 expression in BMFs, Despite the use of ICBs has dramatically increased the survival periods of tumor patients, the response rate was still relatively low in most of the cancer types. Meanwhile, a large number of patients were found resistant to initial dose of treatment during the process of the treatment (47–49). These facts suggest that it is imperative to develop combination strategy to extend the beneficiaries from the ICB immunotherapy and eliminate resistance to these therapy strategies. As illustrated in **Figure 5**, the additional inhibition of Wnt/β-catenin signaling facilitated the anti-tumor immune responses and boosted the efficacy of aPD-L1 immunotherapy in BMF-rich tumors.

In conclusion, BMF-rich tumors had relatively poor response to PD-L1 blockade immunotherapy and the attenuated anti-tumor immune responses was attributed to the expression of PD-L1 on BMFs. The crosstalk between tumor cells and BMFs induced the upregulation of PD-L1 in BMFs by activating the Wnt/β-catenin signaling pathway in BMFs, which was abrogated by Wnt/β-catenin signaling inhibitors. Our findings offer novel insights into the mechanism by which BMFs mediated tumor resistance to immunotherapy and provide potential therapy target to boost therapeutic outcome of immunotherapy *via* interrupting connections between BMFs and cancer cells.

## Data Availability Statement

The raw data supporting the conclusions of this article will be made available by the authors, without undue reservation.

## Ethics Statement

The animal study was reviewed and approved by Institution of Animal Care and Use Committee of Renji Hospital affiliated to Shanghai Jiaotong University School of Medicine.

## Author Contributions

TH and FL designed experiments, analyzed data, and wrote the manuscript. TH, FL, XC, JW, WZ, BZ, YT, QL, and CZ performed the experiments. ST provided overall scientific guidance and supervised the scientific work. All authors contributed to the article and approved the submitted version. All authors contributed to the article and approved the submitted version.

## Funding

This work was financially supported by the National Natural Science Foundation of China (NSFC 81472727, NSFC 81773259 and NSFC 91029718); Science and Technology Commission of Shanghai Municipality (15JC1403100); National laboratory of Oncogene and Cancer- related Genes foundation (90-15-05). Science and Technique Foundation of Henan Province (No. 202102310121 for JW).

## Conflict of Interest

The authors declare that the research was conducted in the absence of any commercial or financial relationships that could be construed as a potential conflict of interest.
