# Supplementary material for: Wnt Inhibition Sensitizes PD-L1 Blockade Therapy by Overcoming Bone Marrow-Derived Myofibroblasts-Mediated Immune Resistance in Tumors
Source: Front Immunol. 2021 Mar 15;12:619209. doi: 10.3389/fimmu.2021.619209 (PMC8006364; doi:10.3389/fimmu.2021.619209)
Supplement: Supplementary file 1 [file Image_1.pdf]

Supplementary Material

Supplementary Figures

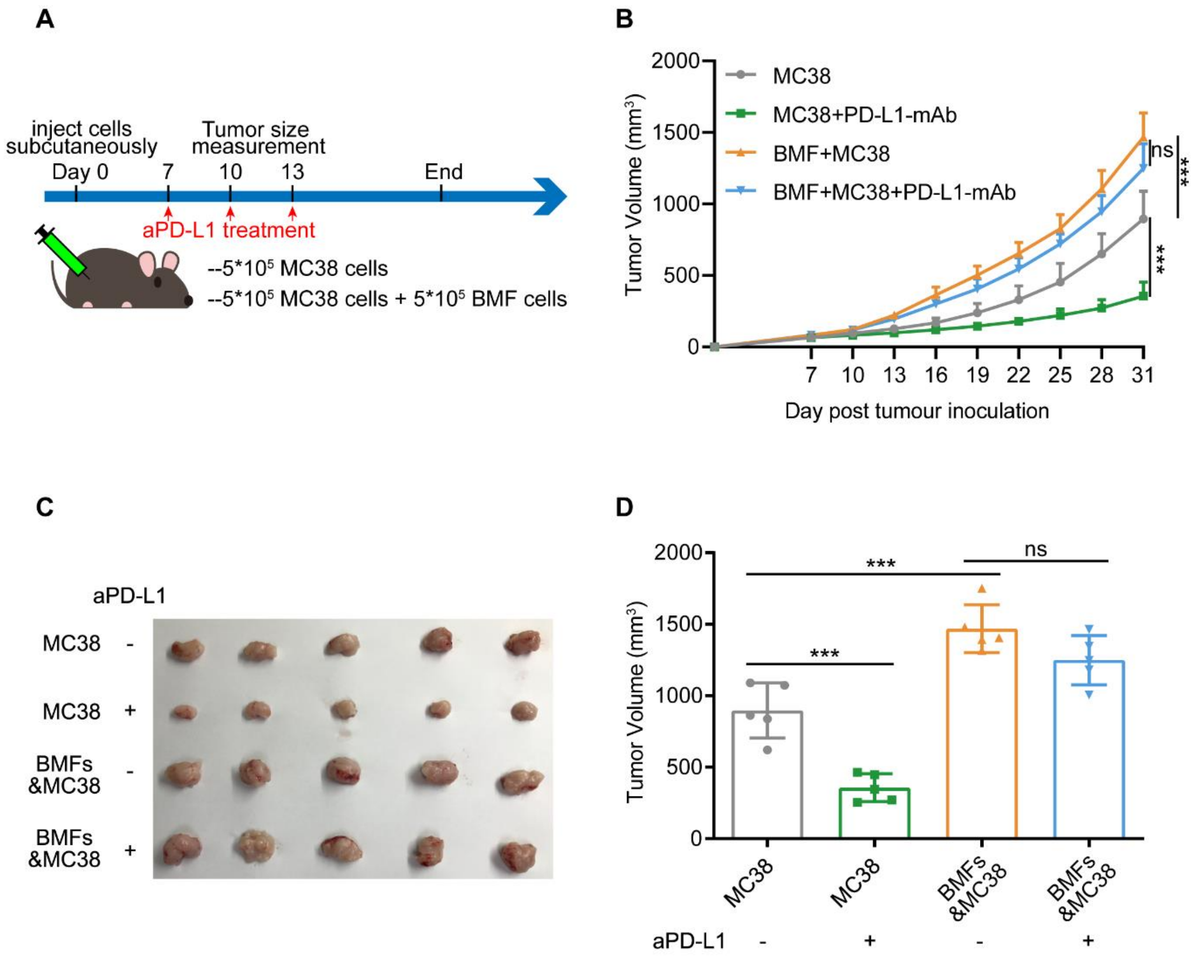

Supplementary Figure 1 BMFs promote resistance to aPD-L1 immunotherapy. **(A)** Schematic diagram for the therapeutic regimen of anti-PD-L1 antibody in tumor-bearing mice injected subcutaneously with MC38 cells ($5*10^5$) alone or mixed with BMFs ($5*10^5$). **(B-D)** Average tumor growth curves**(B)**, the images of tumor tissue dissected from animals**(C)** as well as individual tumor volume **(D)** at the end of the study. The data were plotted as means ± SDs (n = 5 per group) and analyzed using two-tailed Student's t-test for the comparisons between two groups and one-way analysis of variance (ANOVA) with Tukey’s post hoc analysis for multiple comparisons. Differences were considered statistically significant when $P < 0.05$ (***$p < 0.001$, ns stands for not significant).

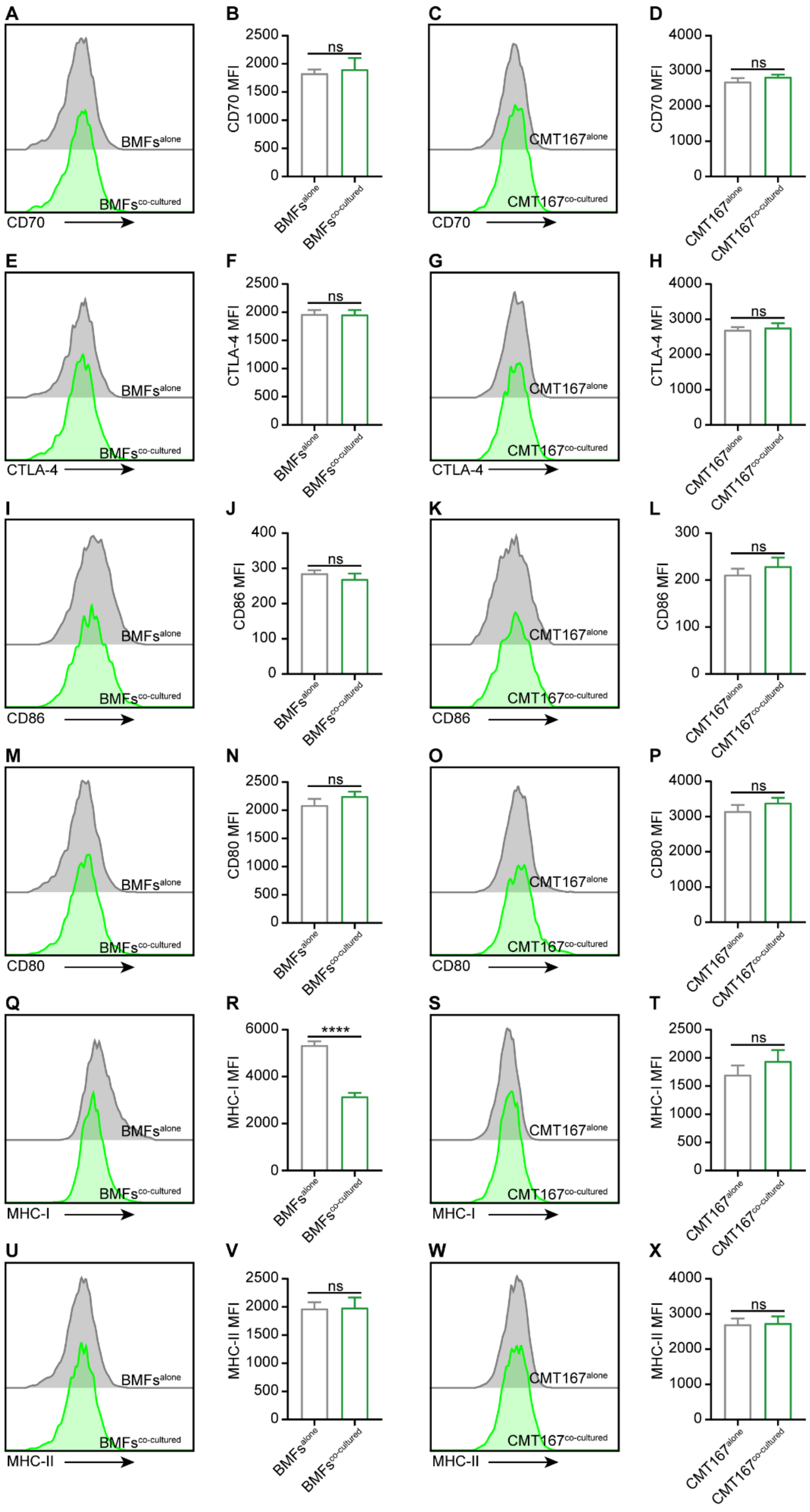
A
B
C
D
E
F
G
H
I
J
K
L
M
N
O
P
Q
R
S
T
U
V
W
X
BMFs alone
BMFs co-cultured
CMT167 alone
CMT167 co-cultured
CD70
CTLA-4
CD86
CD80
MHC-I
MHC-II
CD70 MFI
CTLA-4 MFI
CD86 MFI
CD80 MFI
MHC-I MFI
MHC-II MFI
ns
****

Supplementary Figure 2 Representative histograms and quantitative analysis displaying the expression of CD70 **(A-D)**, CTLA-4 **(E-H)**, CD86 **(I-L)**, CD80 **(M-P)**, MHC-I **(Q-T)**, and MHC-II **(U-X)** in BMFs or CMT167 cells cultured alone or co-cultured with the other. MFI, mean fluorescence intensity. The data were plotted as means ± SDs of three independent experiments (n = 3) and analyzed using two-tailed Student's t-test for the comparisons between two groups and one-way analysis of variance (ANOVA) with Tukey's post hoc analysis for multiple comparisons. Differences were considered statistically significant when P < 0.05 (****p < 0.0001, ns stands for not significant).

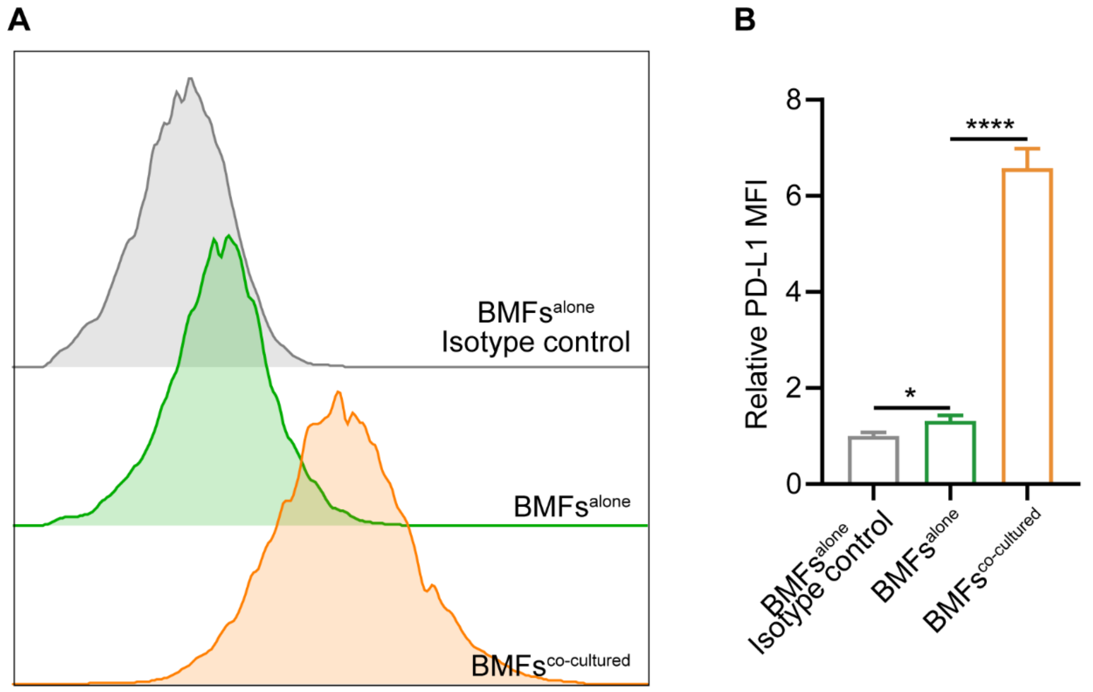

Supplementary Figure 3 Representative histograms **(A)** and quantitative analysis **(B)** displaying the expression level of PD-L1 in BMFs cultured alone or co-cultured with MC38. The data were plotted as means ± SDs (n = 3 per group). MFI, mean fluorescence intensity. The data were analyzed using two-tailed Student's t-test for the comparisons between two groups and one-way analysis of variance (ANOVA) with Tukey's post hoc analysis for multiple comparisons. Differences were considered statistically significant when P < 0.05 (*p < 0.05, ****p < 0.0001. ns stands for not significant).

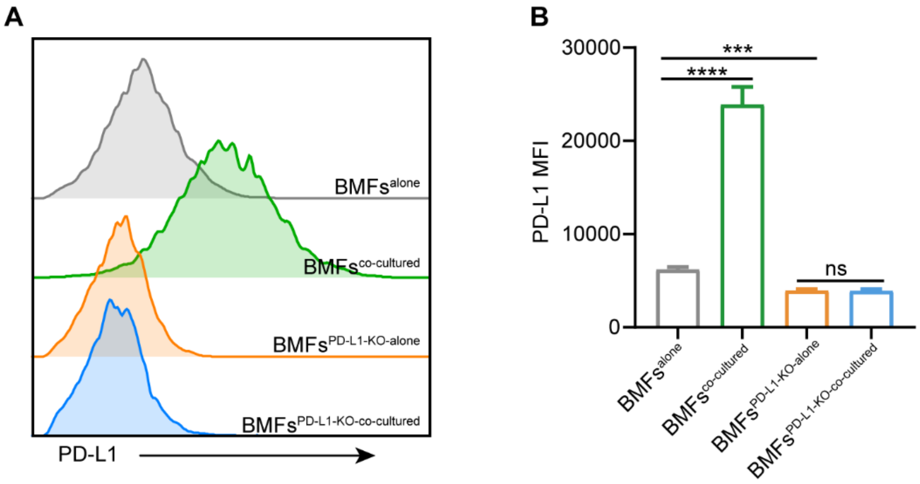

Supplementary Figure 4 Representative histograms **(A)** and quantitative analysis **(B)** displaying the expression level of PD-L1 in BMFs and BMFs$^{PD\text{-}L1\text{-}KO}$ cultured alone or co-cultured with CMT167. MFI, mean fluorescence intensity. The data were plotted as means ± SDs of three independent experiments (n = 3) and were analyzed using two-tailed Student's t-test for the comparisons between two groups and one-way analysis of variance (ANOVA) with Tukey's post hoc analysis for multiple comparisons. Differences were considered statistically significant when $P < 0.05$ (***$p < 0.001$, ****$p < 0.0001$. ns stands for not significant).

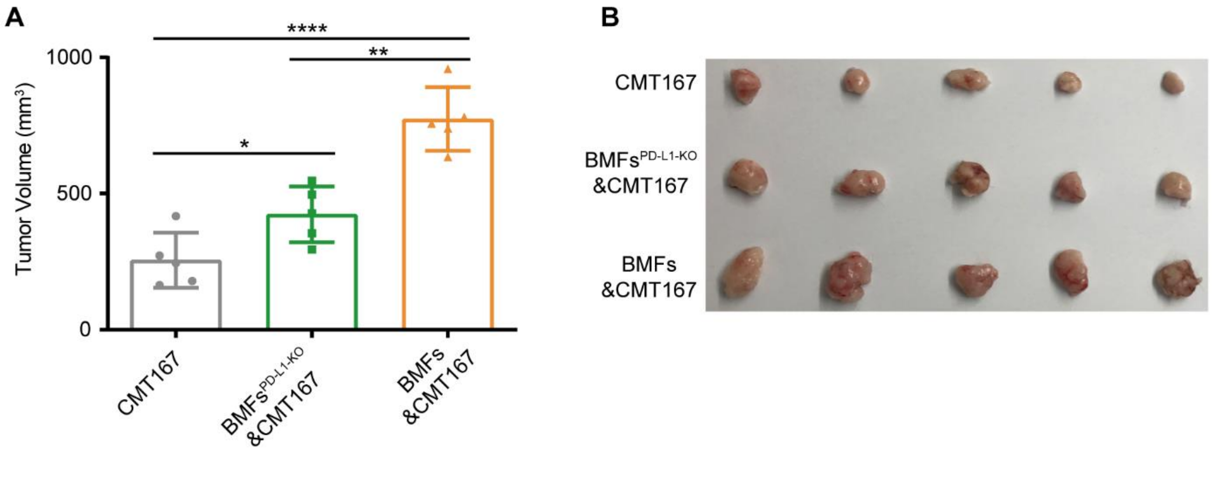

Supplementary Figure 5 Individual tumor volume **(A)** and the images of tumor tissue **(B)** dissected from mice subcutaneously injected with CMT167 cells ($5*10^5$) alone or together with BMFs ($5*10^5$) or $BMFs^{PD\text{-}L1\text{-}KO}$ ($5*10^5$) at the end of the study. The data were plotted as means ± SDs (n = 5 per group). The data were analyzed using two-tailed Student's t-test for the comparisons between two groups and one-way analysis of variance (ANOVA) with Tukey's post hoc analysis for multiple comparisons. Differences were considered statistically significant when $P < 0.05$ (*$p < 0.05$, **$p < 0.01$, and ****$p < 0.0001$).

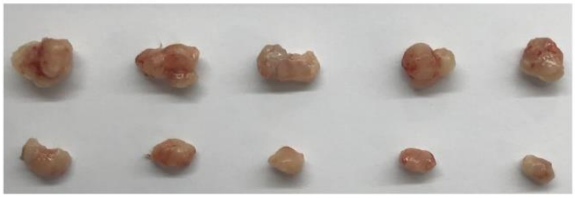

Supplementary Figure 6 The images of tumor tissue dissected from mice at the end of the study. The mice were subcutaneously injected with CMT167 cells ($5*10^5$) mixed with $BMFs^{PD\text{-}L1\text{-}KO}$ ($5*10^5$), followed by treatments with isotype control antibody or aPD-L1 antibody as illustrated in **Figure 3A**.

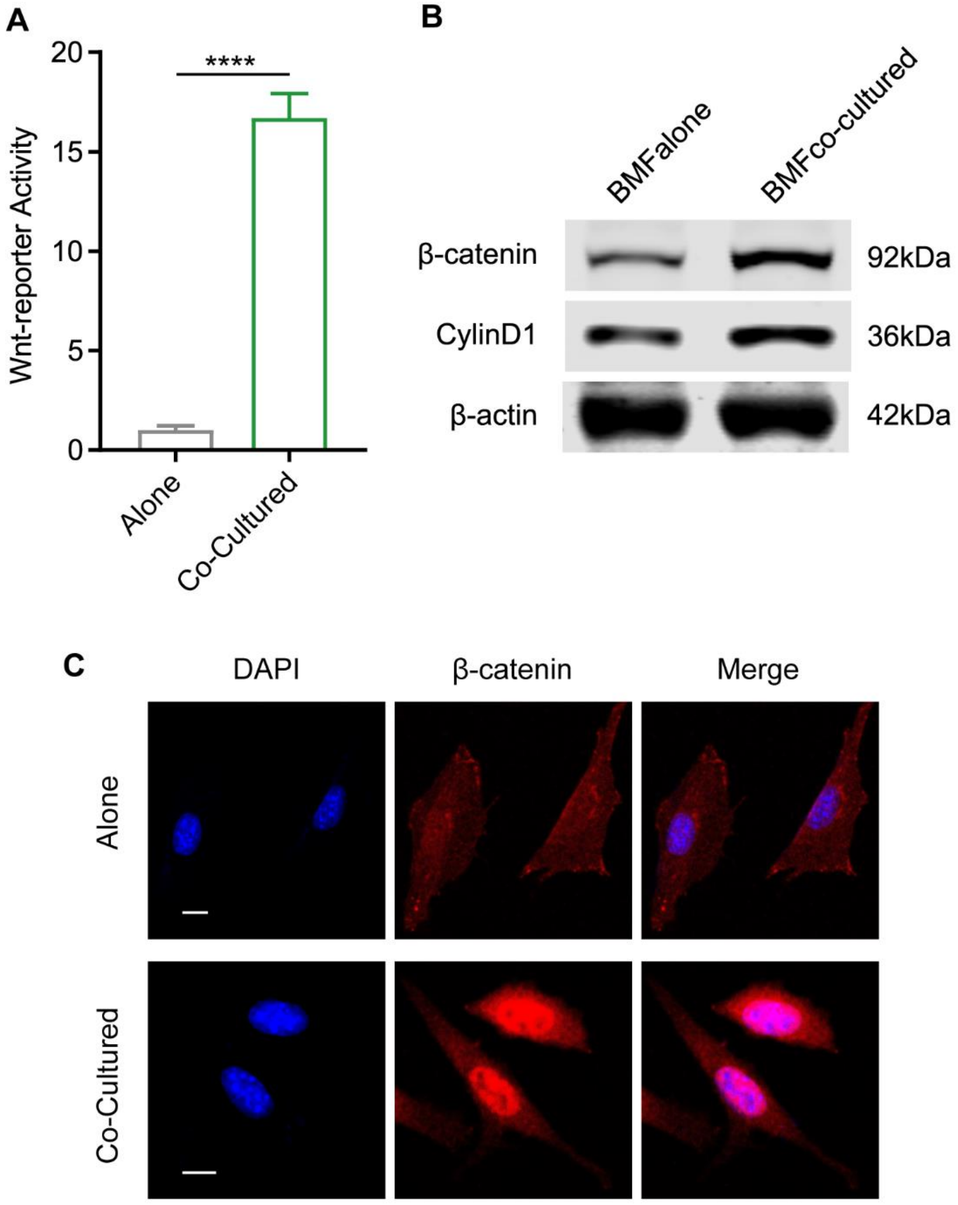

Supplementary Figure 7 (A) Relative Wnt reporter activity of BMFs cells cultured alone or co-cultured with MC38. **(B)** Representative immunoblotting result of β-catenin and CylinD1 in BMFs cultured alone or co-cultured with MC38. β-actin was used as the loading control. **(C)** Representative image of β-catenin (red) immunofluorescence staining in BMFs cultured alone or co-cultured with MC38. DAPI was stained to visualize cell nuclei. Scale bar = 10 μm. Representative results were from one of at least three independent experiments. The data were plotted as means ± SDs (n = 3 per group) and analyzed using two-tailed Student's t-test for the comparisons between two groups and one-way analysis of variance (ANOVA) with Tukey's post hoc analysis for multiple comparisons. Differences were considered statistically significant when $P < 0.05$ (****$p < 0.0001$).

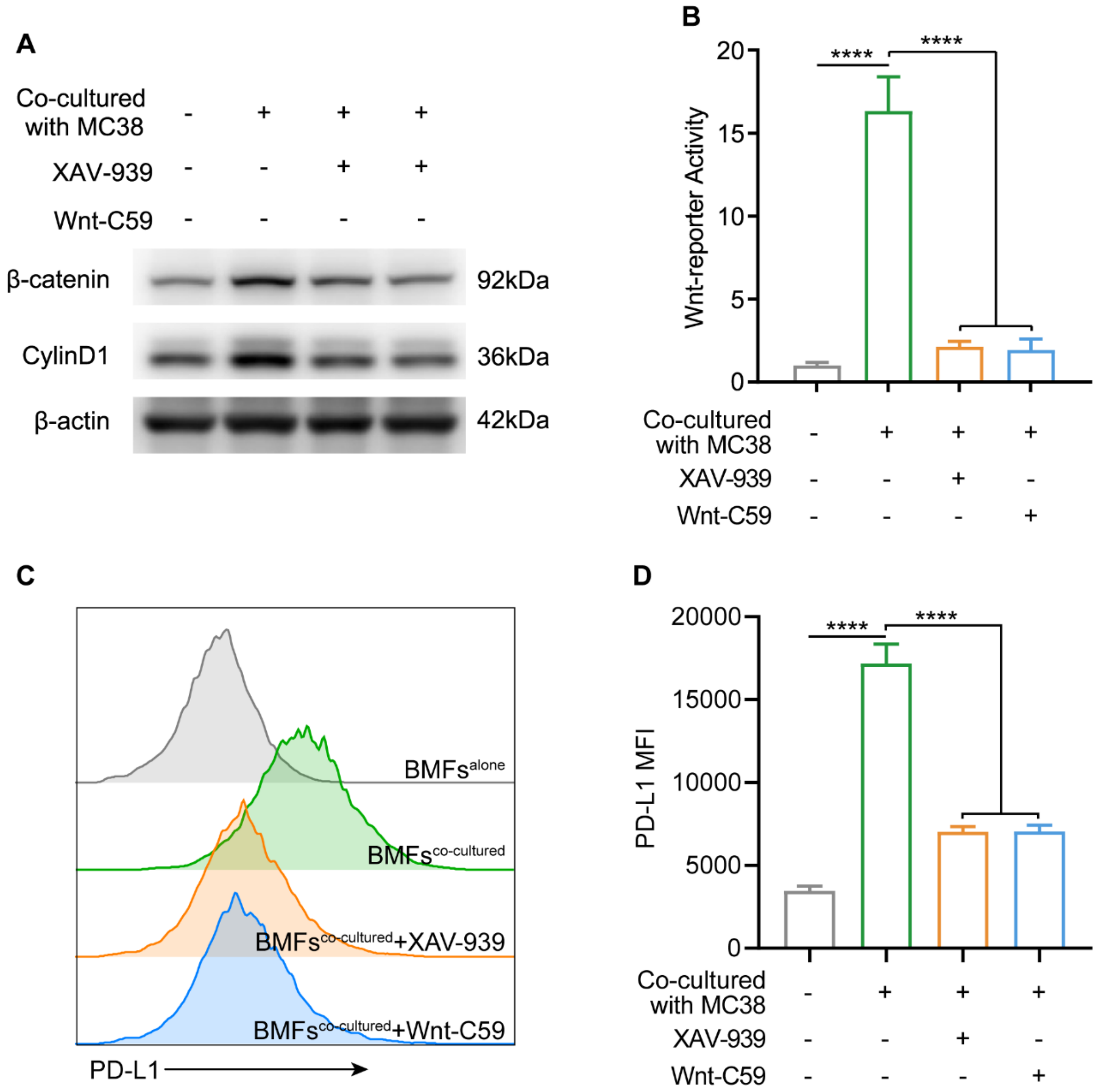

Supplementary Figure 8 Relative wnt reporter activity**(A)**, representative immunoblotting result of β-catenin, CylinD1 and β-actin**(B)** and PD-L1 express level measured by flow cytometry **(C, D)** in BMFs cells cultured alone or co-cultured with MC38 while the co-cultured group treated with XAV-939(1μM)/Wnt-C59(5μM) or not. MFI, mean fluorescence intensity. Representative results were from one of at least three independent experiments. The data were plotted as means ± SDs (n = 3 per group) and analyzed using two-tailed Student's t-test for the comparisons between two groups and one-way analysis of variance (ANOVA) with Tukey's post hoc analysis for multiple comparisons. Differences were considered statistically significant when $P < 0.05$ (****$p < 0.0001$).

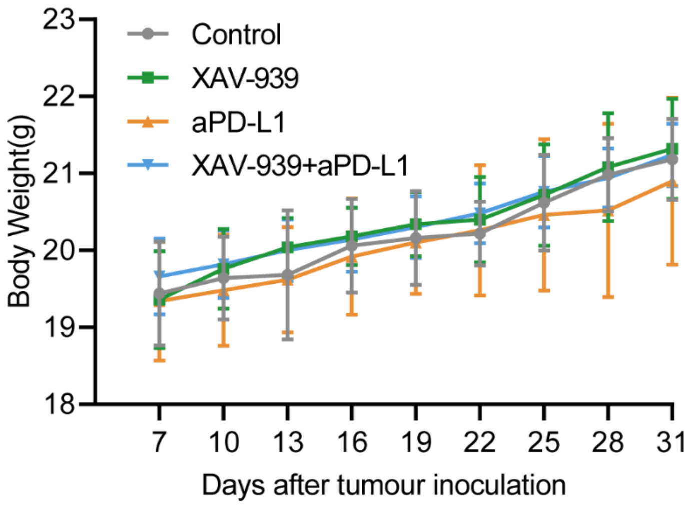

Supplementary Figure 9 Mice body weight among the entire therapeutic study of aPD-L1 antibody combined with XAV-939 in tumor-bearing mice injected subcutaneously with CMT167 cells ($5*10^5$) together with BMFs ($5*10^5$) as illustrated in **Figure 5A**. The data were plotted as means ± SDs (n = 5 per group). The data were analyzed using two-tailed Student's t-test for the comparisons between two groups and one-way analysis of variance (ANOVA) with Tukey's post hoc analysis for multiple comparisons. Differences were considered statistically significant when $P < 0.05$.
